# Public knowledge of thyroid disorders in the West Bank, Palestine: pregnancy and medication-related safety gaps in a cross-sectional study

**DOI:** 10.3389/fpubh.2026.1798507

**Published:** 2026-03-31

**Authors:** Amani B. Ahmed, Amal Obeid, Abdullah Abukeshek, Mohammad Hakam Shehadeh, Mohammed M. Salahaldin, Hamza Karmi, Haitham Suwan, Ahmed Salama, Sohaib Dwaik, Hobab Odeh, Nuha Riyad, Osama Ewidat, Ameer B. Ahmed, Mohammed Maree

**Affiliations:** 1 Department of General Surgery, Al-Makassed Islamic Charitable Hospital, East Jerusalem, Palestine; 2 Faculty of Medicine, Al-Quds University, Jerusalem, Palestine; 3 Department of Urology, Hebron Governmental Hospital, Hebron, Palestine; 4 General Medicine, Al-Istishari Arab Hospital, Ramallah, Palestine; 5 Department of Medicine, Faculty of Medicine and Health Sciences, An-Najah National University, Nablus, Palestine

**Keywords:** medication safety, Palestine, pregnancy and postpartum, public knowledge, thyroid disorders

## Abstract

**Background:**

Thyroid disorders are common and often under-recognized. Evidence on public thyroid health literacy in Palestine is limited. This study assessed public knowledge of thyroid disorders among adults in the West Bank, Palestine.

**Methods:**

A cross-sectional online survey was conducted from May 1 to August 1, 2025, among adults (≥18 years) residing in the West Bank. Participants with current or prior health-professional training or employment in healthcare were excluded. Non-probability convenience sampling was used through social media distribution (Facebook, WhatsApp, Telegram). Knowledge was assessed using a 17-item multiple-choice instrument covering risk factors (7 items), clinical manifestations (7 items), and preventive behaviors (3 items). Correct responses were scored as 1 and incorrect/“I don’t know” as 0; knowledge levels were categorized as low (<50%), moderate (50–75%), and high (>75%). Data were analyzed using descriptive statistics, chi-square tests, and ordinal logistic regression.

**Results:**

A total of 1,119 participants were included (71.3% women, 75.0% university-educated). Mean knowledge score was 8.60/17 (50.6%); 56.7% had low knowledge, 26.3% moderate, and 17.1% high. Risk-factor knowledge was lowest (mean 2.97/7; 42.4%), while prevention-related knowledge was comparatively higher (mean 2.03/3; 67.7%). Notable gaps were observed for medication-related risks (amiodarone 9.7%; lithium 11.0%) and pregnancy/postpartum risk (41.7%). In adjusted analyses, higher knowledge was associated with age >50 years (aOR 1.71), while lower knowledge was associated with male sex (aOR 0.70) and no formal education versus university education (aOR 0.26).

**Conclusion:**

Public knowledge of thyroid disorders in the West Bank was generally low, with the most pronounced deficits related to pregnancy and the postpartum period and to medication associated thyroid dysfunction. These findings support targeted, low cost health education integrated into routine primary care, maternal and reproductive health services, and community pharmacy counseling, with future studies needed to evaluate effects on timely care seeking and safer medication practices.

## 1 Introduction

Endocrine disorders, especially those affecting the thyroid, have become increasingly prevalent over time. The thyroid gland, which resembles a butterfly and is located in the neck, plays a crucial role in regulating metabolism, growth, and development through the ongoing release of thyroid hormones (1). Thyroid problems are very common: estimates suggest on the order of 200 million people worldwide have some form of thyroid disorder (2). For example, overt hypothyroidism affects roughly 5% of the population (with another ~5% undiagnosed) (3), and thyroid nodules or goiter may be found in a similar magnitude at autopsy. Key risk factors include iodine deficiency, which can cause endemic goiter and hypothyroidism, female sex and age (women are at least four times more likely than men to develop thyroid disorders (4), and prevalence rises in older age), and autoimmune predisposition (with family history or comorbid autoimmunity). Environmental exposures (for example, childhood radiation) and certain medications also increase risk. Untreated thyroid disease has broad impacts: hypothyroidism can cause fatigue, weight gain, cold intolerance and, over time, raise cardiovascular risk (3), while hyperthyroidism can produce weight loss, palpitations, nervousness and bone loss. Both can impair fertility and quality of life (3).

In public health terms, early detection of thyroid disease critically depends on public awareness of symptoms and risk factors. Because symptoms (for example, fatigue, weight changes, mood or menstrual disturbances) are often nonspecific, people with low knowledge may delay seeking care. Studies in the region show that this is indeed a problem: for instance, a Saudi survey found that nearly half the population scored poorly on thyroid knowledge (5), and a Jordanian survey revealed major gaps in recognizing women’s health-related symptoms of thyroid dysfunction (6). Inadequate knowledge “frequently leads patients to go undiagnosed” (5). Conversely, better-informed individuals are more likely to engage in preventive behaviors; the Jordan study found that perceiving thyroid disorders as preventable correlated with higher knowledge scores (6). Public education has been shown to improve early detection in other contexts. For example, a study in Northern Saudi Arabia documented low public awareness of thyroid cancer risk factors and screening (with 54.6% never having a thyroid test), and concluded that “culturally tailored educational interventions” are needed to improve outcomes (7). These findings underscore that improving thyroid health literacy and awareness of when to test or treat can lead to timelier diagnosis and management, reducing complications.

Palestine faces unique socio-economic and healthcare challenges that affect thyroid disease management. Politically, movement restrictions and conflict have fragmented the health system. Recent reports describe thousands of Palestinians (especially in Gaza) lacking access to basic care due to destroyed clinics and “shortage of essential medications… and diagnostic equipment” (8). The Israeli occupation’s permit regime, internal closures and economic hardships also hinder routine care. These constraints make early screening for chronic diseases, including thyroid disorders, very difficult. Socio-cultural factors add further complexity: although general literacy is high in Palestine (adult illiteracy ~2%), health literacy may lag and is uneven across regions and populations. Poverty (24% nationally) and high unemployment (especially among women) limit people’s ability to seek non-urgent care (9). Importantly, no published study has examined thyroid disease awareness in Palestine, which is a critical gap given the region’s disease burden. Notably, recent surveys indicate adequate iodine nutrition in the West Bank, suggesting that most thyroid dysfunction here is due to other causes (10).

In short, Palestinians face the combined effects of constrained healthcare infrastructure and low disease awareness, which likely delay diagnosis and treatment of thyroid conditions. Generating locally grounded evidence on thyroid health literacy is therefore not merely descriptive; it can identify specific misconceptions that are most actionable for low-cost interventions (for example, targeted counseling prompts in primary care, maternal health services, and pharmacy encounters). Accordingly, this study aimed to quantify public knowledge regarding thyroid disorders among adults in the West Bank, to characterize the most prominent misconceptions with emphasis on pregnancy/postpartum and medication-related risks, and to examine sociodemographic factors associated with higher knowledge to inform targeted, context-appropriate public health messaging.

## 2 Methods

### 2.1 Study design and population

This was a cross-sectional study designed to assess public awareness and knowledge of thyroid diseases in Palestine. The study was conducted from May 1 to August 1, 2025.

### 2.2 Study population and sampling

The target population consisted of adults aged 18 years and above residing in the West Bank regardless of personal history of thyroid disorders. Participants who reported current or previous formal health professional training (medical students, nursing/allied health students) or employment in healthcare (for example, physicians, nurses, pharmacists, laboratory staff, allied health professionals) were excluded to better reflect knowledge levels in the general population and to minimize information bias due to professional exposure to thyroid-related content.

Non-probability convenience sampling was conducted via social media platforms (Facebook, WhatsApp, and Telegram). Sample size calculation was performed using OpenEpi (11) with 50% expected frequency, 95% confidence level, and 5% margin of error, yielding a minimum required sample of 384 participants. However, to enhance statistical power, a 1,119 completed questionnaires were included in the final analysis. Given the online, non-probability recruitment approach, selection bias is possible, with disproportionate participation from individuals with higher educational attainment, greater health awareness, and more frequent engagement with social media platforms.

### 2.3 Data collection instruments

The questionnaire was adopted with permission from validated instruments used in previous research, specifically from the study by Alyahya et al. (5) conducted in Saudi Arabia. The adaptation process involved review by experts in endocrinology, public health, and epidemiology and pilot testing of 30 participants to ensure clarity, relevance, and cultural appropriateness. The questionnaire was administered in Arabic. Our analyses indicated adequate internal consistency with Cronbach’s *α* > 0.7 in respective sections.

The questionnaire comprised two primary sections. The first section collected participant characteristics (sex, age group, marital status, education level, family history of thyroid disease, and monthly household income).

Monthly household income was recorded in pre-specified categories (<900, 900–1,800, 1,800–3,000, and >3,000 USD), defined *a priori* to yield interpretable socioeconomic strata and ensure adequate cell sizes for regression analyses. The lowest category (<900 USD) was selected to approximate households near the nationally used poverty threshold (≈705 USD/month for a reference household of five) (12).

The second section assessed participants’ knowledge of thyroid disorders through 17 multiple-choice questions grouped into three domains. The first domain assessed risk factors using 7 items (Cronbach’s *α* = 0.867). The second domain evaluated clinical manifestations through 7 items (Cronbach’s *α* = 0.885). The final domain examined preventive behaviors using 3 items (Cronbach’s *α* = 0.731). The correct responses were scored as one point and incorrect or “I don’t know” responses scored as zero. Knowledge levels were categorized as low (less than 50%), moderate (50 to 75%), and high (more than 75%). These thresholds were defined *a priori* and aligned with the source instrument’s categorization (0–8 low, 9–12 moderate, 13–17 high).

### 2.4 Data collection procedure

Data were collected using an anonymous, self-administered online questionnaire via Google Forms. Recruitment messages containing the study invitation and survey link were disseminated across multiple social media channels including Facebook groups, WhatsApp groups, and Telegram channels. The first page of the survey included an eligibility screen (age ≥18 years; West Bank residency; no current or prior formal health-professional training or healthcare employment); individuals who did not meet eligibility criteria were instructed not to proceed. No incentives were offered for participation.

### 2.5 Statistical analysis

Data were exported to Jamovi statistical software (version 2.6.44) for analysis. Descriptive statistics were presented as frequencies and percentages for categorical variables and means with standard deviations for continuous variables.

Normality of continuous data were assessed using Kolmogorov–Smirnov test, along with plot visualization, which indicated a non-normal distribution for most numerical variables. Nonetheless, means (SD) were presented due to the narrow range of scales and for better comparison, as median and interquartile range were less informative in this context.

Knowledge scores were calculated by summing correct responses. Bivariate associations between knowledge categories and independent variables (such as age, sex, education) were evaluated using chi-square tests. Ordinal logistic regression was performed to identify independent predictors of adequate thyroid disease knowledge. Statistical significance was set at *p* < 0.05,

### 2.6 Ethical considerations

Ethical approval was obtained from Al-Quds University Research Ethics Committee (Ref No: 542/REC/2025). Before accessing the questionnaire, participants were presented with a section that provided clear and comprehensive information regarding the study’s aims, their voluntary participation, and data confidentiality, with electronic informed consent obtained before proceeding to the questionnaire. All collected data were anonymized and stored securely, with access restricted exclusively to the research team.

## 3 Results

### 3.1 Sample characteristics

A total of 1,119 adults completed the survey; 71.3% were Female and 75.0% had university-level education. Age was recorded as a categorical variable (18–25, 26–50, and >50 years). The mean total knowledge score was 8.60 (SD 3.90) out of 17, and 56.7% of participants were classified as having poor knowledge (<50% of the maximum score). Participant characteristics and knowledge categories are presented in Table 1.

**Table 1:** Sociodemographic characteristics and knowledge score of participants (*N* = 1,119).

Variables	Count	%
Gender		
Women	798	(71.3%)
Men	321	(28.7%)
Age		
18–25	301	(26.9%)
26–50	568	(50.8%)
More than 50	250	(22.3%)
Marital status		
Single	320	(28.6%)
Married	698	(62.4%)
Divorced	44	(3.9%)
Widowed	57	(5.1%)
Education level		
No education	21	(1.9%)
Primary	79	(7.1%)
Secondary	180	(16.1%)
University	839	(75.0%)
Monthly household income (USD)		
Less than 900	228	(20.4%)
900–1,800	584	(52.2%)
1,800–3,000	249	(22.3%)
More than 3,000	58	(5.2%)
Family history of thyroid disease		
Yes	374	(33.4%)
No	745	(66.6%)
Knowledge score		
Mean (± SD)	8.60 + 3.898	
Knowledge score percentile		
Low (<50%)	634	56.7%
Moderate (50–75%)	294	26.3%
High (>75%)	191	17.1%

### 3.2 Assessment of knowledge regarding risk factors, manifestations, and preventive behaviors of participants (*N* = 1,119)

At the item level, recognition of risk factors was highest for female sex (67.6%) and smoking (65.2%), while awareness of pregnancy and postpartum risk was lower (41.7%). Knowledge of medication-related risks was particularly limited, with 9.7 and 11.0% correctly identifying amiodarone and lithium, respectively. Among clinical manifestations, a neck lump (81.1%) and fatigue (78.6%) were most frequently recognized. Item-level responses are reported in Table 2.

**Table 2:** Assessment of knowledge regarding risk factors, manifestations, and preventive behaviors of participants.

Domain	Item	Yes, n (%)	No, n (%)	Do not know, n (%)
Risk factors	Smoking is a risk factor for thyroid disorders.	730 (65.2%)	88 (7.9%)	301 (26.9%)
Risk factors	Radiation exposure is a risk factor for thyroid disorders.	587 (52.5%)	98 (8.8%)	434 (38.8%)
Risk factors	Females are at higher risk of thyroid disorders.	757 (67.6%)	62 (5.5%)	300 (26.8%)
Risk factors	Insufficient or excess iodine intake is a risk factor for thyroid disorders.	549 (49.1%)	76 (6.8%)	494 (44.1%)
Risk factors	Pregnancy and the postpartum period are risk factors for thyroid disorders.	467 (41.7%)	148 (13.2%)	504 (45.0%)
Risk factors	Amiodarone use is a risk factor for thyroid disorders.	108 (9.7%)	61 (5.5%)	950 (84.9%)
Risk factors	Lithium use is a risk factor for thyroid disorders.	123 (11.0%)	59 (5.3%)	937 (83.7%)
Clinical manifestations	Cold intolerance and weight gain are common symptoms of hypothyroidism.	479 (42.8%)	34 (3.0%)	606 (54.2%)
Clinical manifestations	Heat intolerance and weight loss are common symptoms of hyperthyroidism.	447 (39.9%)	23 (2.1%)	649 (58.0%)
Clinical manifestations	A neck lump can be a sign of thyroid disorders.	908 (81.1%)	53 (4.7%)	158 (14.1%)
Clinical manifestations	Fatigue can be a symptom of thyroid disorders.	879 (78.6%)	53 (4.7%)	187 (16.7%)
Clinical manifestations	Diarrhea, constipation, or abdominal pain can be symptoms of thyroid disorders.	451 (40.3%)	174 (15.5%)	494 (44.1%)
Clinical manifestations	Skin and nail changes or hair loss can be signs of thyroid disorders.	404 (36.1%)	173 (15.5%)	542 (48.4%)
Clinical manifestations	Bulging eyes can be a sign of thyroid disorders.	464 (41.5%)	148 (13.2%)	507 (45.3%)
Prevention	Avoiding soy-based foods is a preventive measure for thyroid disorders in women.	417 (37.3%)	77 (6.9%)	625 (55.9%)
Prevention	Early thyroid function testing can prevent complications of thyroid disorders.	907 (81.1%)	49 (4.4%)	163 (14.6%)
Prevention	A well-balanced diet is essential for preventing thyroid disorders.	854 (76.3%)	70 (6.3%)	195 (17.4%)

### 3.3 Knowledge domain scores

Domain-level performance was lowest for risk-factor knowledge (mean 2.97 out of 7; 42.4% of the maximum), whereas prevention-related knowledge was comparatively higher (mean 2.03 out of 3; 67.7% of the maximum). Domain scores are summarized in Table 3.

**Table 3:** Knowledge domain scores.

Domain	Items	Possible range	Mean (SD)	Mean % of maximum	Observed range
Knowledge regarding risk factors	7	0–7	2.97 (1.87)	42.4	0–7
Knowledge regarding manifestations	7	0–7	3.60 (1.85)	51.4	0–7
Knowledge regarding preventive behaviors	3	0–3	2.03 (0.98)	67.7	0–3
Total knowledge score	17	0–17	8.60 (3.90)	50.6	0–17

### 3.4 Bivariate associations with thyroid knowledge category

As shown in Table 4, Knowledge category differed by age group (*p* = 0.033), marital status (*p* = 0.005), monthly household income (*p* < 0.001), and educational level (*p* < 0.001), whereas no association was observed with gender (*p* = 0.159). Participants aged >50 years constituted a larger proportion of the high-knowledge group than of the poor-knowledge group (29.8% vs. 20.7%). University education was more prevalent among participants with high knowledge (86.4%) than among those with poor knowledge (68.3%). Higher income (1800–3,000 USD/month) was also more common in the high-knowledge group (34.0%) compared with the poor-knowledge group (17.2%), while the <900 USD category was less represented among high knowledge (11.5% vs. 23.7%).

**Table 4:** Participant characteristics by thyroid knowledge category and chi-square comparisons (*N* = 1,119).

Characteristic	Total (*N* = 1,119)	Poor knowledge (<50%) (*n* = 634)	Moderate knowledge (50–75%) (*n* = 294)	High knowledge (>75%) (*n* = 191)	*p*-value
Age, years					**0.033**
18–25	301 (26.9%)	186 (29.3%)	75 (25.5%)	40 (20.9%)	
26–50	568 (50.8%)	317 (50.0%)	157 (53.4%)	94 (49.2%)	
>50	250 (22.3%)	131 (20.7%)	62 (21.1%)	57 (29.8%)	
Gender					0.159
Women	798 (71.3%)	440 (69.4%)	212 (72.1%)	146 (76.4%)	
Men	321 (28.7%)	194 (30.6%)	82 (27.9%)	45 (23.6%)	
Marital status					**0.005**
Single	320 (28.6%)	202 (31.9%)	77 (26.2%)	41 (21.5%)	
Married	698 (62.4%)	386 (60.9%)	186 (63.3%)	126 (66.0%)	
Divorced	44 (3.9%)	14 (2.2%)	17 (5.8%)	13 (6.8%)	
Widowed	57 (5.1%)	32 (5.0%)	14 (4.8%)	11 (5.8%)	
Monthly household income (USD)					**<0.001**
<900	228 (20.4%)	150 (23.7%)	56 (19.0%)	22 (11.5%)	
900–1,800	584 (52.2%)	340 (53.6%)	150 (51.0%)	94 (49.2%)	
1,800–3,000	249 (22.3%)	109 (17.2%)	75 (25.5%)	65 (34.0%)	
>3,000	58 (5.2%)	35 (5.5%)	13 (4.4%)	10 (5.2%)	
Educational level					**<0.001**
No formal education	21 (1.9%)	16 (2.5%)	1 (0.3%)	4 (2.1%)	
Primary education	79 (7.1%)	61 (9.6%)	11 (3.7%)	7 (3.7%)	
Secondary education	180 (16.1%)	124 (19.6%)	41 (13.9%)	15 (7.9%)	
University	839 (75.0%)	433 (68.3%)	241 (82.0%)	165 (86.4%)	
Family history of thyroid disease					0.530
Yes	374 (33.4%)	210 (33.1%)	105 (35.7%)	59 (30.9%)	
No	745 (66.6%)	424 (66.9%)	189 (64.3%)	132 (69.1%)	

Values are n (column %). Knowledge categories were defined as poor (<50%), moderate (50–75%), and high (>75%). Bold *p*-values indicate statistically significant associations at *p* < 0.05.

### 3.5 Ordinal logistic regression for factors associated with higher thyroid knowledge level

The logistic regression model was statistically significant, χ^2^(12) = 96.5, *p* < 0.001. Pseudo-*R*^2^ values indicated modest explanatory performance (McFadden’s *R*^2^ = 0.0442; Cox and Snell *R*^2^ = 0.0283; Nagelkerke *R*^2^ = 0.0593; *N* = 1,119). No problematic multicollinearity was detected among predictors (VIF range = 1.03–1.18; maximum VIF = 1.18).

As presented in Table 5, respondents aged >50 years had higher odds of being in a higher thyroid knowledge category compared with those aged 18–25 years (aOR 1.70, *p* = 0.018), whereas the 26–50 group did not differ significantly. Men had lower odds of higher knowledge than women (aOR 0.70, *p* = 0.010). Compared with married participants, those who were divorced showed higher odds of better knowledge (aOR 2.31, *p* = 0.004), while single and widowed participants were not significantly different. Compared with participants with university education, those with no formal education had significantly lower odds of being in a higher knowledge category (aOR 0.26, *p* = 0.014). For monthly household income, the (1800–3,000 category) vs. (900–1800 category) was associated with higher knowledge (aOR 1.61, *p* = 0.001), while (<900 category) and (>3,000 category) were not significantly associated.

**Table 5:** Ordinal logistic regression for factors associated with higher thyroid knowledge level.

Predictor	Category	Adjusted OR	95% CI	*p*-value
Age group	26–50 vs. 18–25 (ref)	1.104	0.7865–1.552	0.567
	>50 vs. 18–25 (ref)	1.691	1.0937–2.615	0.018
Gender	Men vs. Women (ref)	0.701	0.5341–0.916	0.010
Marital status	Widowed vs. Married (ref)	1.281	0.7232–2.237	0.389
	Single vs. Married (ref)	0.765	0.5460–1.068	0.117
	Divorced vs. Married (ref)	2.314	1.3014–4.111	0.004
Education level	Primary vs. University (ref)	0.238	0.1289–0.422	<0.001
	Secondary vs. University (ref)	0.456	0.3182–0.645	<0.001
	No formal education vs. University (ref)	0.257	0.0787–0.712	0.014
Monthly household income (USD)	<900 vs. 900–1,800 (ref)	0.810	0.5842–1.117	0.202
	1,800–3,000 vs. 900–1800 (ref)	1.613	1.2043–2.160	0.001
	>3,000 vs. 900–1,800 (ref)	0.908	0.5160–1.560	0.731
Family history of thyroid disease	Yes vs. No (ref)	1.040	0.8056–1.341	0.762

## 4 Discussion

Thyroid disorders represent an important public health concern, and this study offers the first regional data on public awareness in the West Bank, Palestine. Overall knowledge was modest (mean score 8.60/17; 50.6%), with 56.7% of respondents classified as having poor knowledge, 26.3% moderate knowledge, and 17.1% high knowledge. Knowledge gaps were most evident for clinically relevant but less readily recognized risk factors, including drug-induced thyroid dysfunction, and for reproductive and pregnancy-related risks, indicating priority targets for health education interventions.

Our results align with and extend a growing body of evidence that public thyroid knowledge is generally low worldwide. In Saudi Arabia, Alhazmi et al. found that only about half of adults had adequate thyroid awareness (13). Likewise, in Riyadh just 22% of participants achieved a “good” knowledge level (14), a figure only slightly higher than our 17.1%. In Jordan, researchers observed moderate awareness (mean 7.2/14, or 51.4%), with many unable to identify key symptoms (for example, only ~38% recognized menstrual changes as a sign) (6). Other regional studies report similar gaps: for example, in the Hail region of Saudi Arabia 59.7% had heard of thyroid disorders but few knew about risk factors or treatments (15). By comparison, even in high-income countries thyroid knowledge is often suboptimal. A review of European and US data notes that 4–7% of adults have undiagnosed hypothyroidism (16), implying that many are simply unaware of their condition. In Northern Cyprus, a community survey showed that 72% had heard of thyroid disease but true understanding was poor: women reported higher familiarity (78% vs. 68% in men) (17). Similarly, a recent Chinese study of pregnant women found “suboptimal” knowledge and inactive practices regarding thyroid health (18). In short, awareness in Palestine appears neither uniquely low nor high; it is comparable to the moderate levels seen in other populations (Middle Eastern or otherwise) where thyroid disorders are common but under-appreciated. The pattern of our results, mainly educated, middle-aged females with only half answering knowledge items correctly is broadly consistent with these published findings (13, 14).

In multivariable analyses, several sociodemographic characteristics were independently associated with thyroid knowledge. Male respondents had lower odds of being in a higher knowledge category than female respondents, whereas participants aged >50 years had higher odds compared with those aged 18–25 years. A socioeconomic gradient was also observed: lower educational attainment was associated with lower odds of higher knowledge relative to university education, and respondents with a monthly household income of USD 1,800–3,000 had higher odds compared with the USD 900–1,800 reference group. Because age and education may be correlated, the multivariable model simultaneously adjusted for both factors when estimating associations. Family history of thyroid disease was not independently associated with knowledge after adjustment, suggesting that informal exposure through relatives may be insufficient in the absence of structured health information.

The low level of thyroid awareness identified has serious public health implications. As multiple sources note, insufficient knowledge often translates into delayed diagnosis and treatment, and thereby greater morbidity. Thyroid symptoms (fatigue, weight changes, and related symptoms) are nonspecific, so patients and even clinicians can miss the diagnosis unless the public is alert to risk factors and signs (16, 19). Indeed, reviews report that roughly 60% of people with thyroid dysfunction worldwide are unaware of it (19), and many cases of hypothyroidism go undetected until complications occur (16). This invisibility means that preventable complications from severe hypothyroid fatigue to cardiac and metabolic issues may accumulate. Consequently, improving awareness is likely to reduce the burden of untreated disease. Notably, early detection and treatment of thyroid disease are “fundamental” to reducing healthcare costs and improving outcomes (19). Thus, our findings highlight a need for public health strategies to educate the population about thyroid disorders, especially targeting the risk factor gaps we found (such as drug-induced causes, pregnancy-associated risks). Health authorities in Palestine should consider thyroid screening in high-risk groups and bolster education so that cases are identified and managed sooner (16).

Several factors likely underlie the knowledge gaps observed. Education and socioeconomic status play a strong role: our highly educated sample (75% university-educated) still had poor knowledge, suggesting that even among the educated public, detailed thyroid information is lacking. Other studies concur: in Jeddah and Riyadh, higher education and employment were correlated with better scores (14, 19), while unemployed and less educated groups showed the lowest awareness (19). Similarly, in Northern Cyprus and China, education level was tightly linked to self-reported thyroid familiarity (17, 18). Gender roles and cultural factors may also contribute. Women are more prone to thyroid disorders, and many surveys (including our own) find more women participating in these studies (13, 17). Women also tended to have higher knowledge, perhaps because of greater health-seeking behavior or exposure during pregnancy (6). Cultural norms might de-prioritize women’s endocrine health in some settings, compounding unawareness of pregnancy-related thyroid risks (1, 19). Healthcare access and delivery are additional barriers. In rural and under-resourced areas of Palestine, thyroid screening and specialist care may be limited, and general practitioners may not emphasize thyroid education. If patients do not routinely undergo thyroid function tests, they may remain unaware of the disease. Finally, we did not measure participants’ sources of thyroid-related information (for example, clinicians, social media, or mass media). However, other studies suggest that mass media and public campaigns are not the dominant sources of thyroid information for many populations (19, 20), supporting the need to embed education within clinical encounters (primary care, antenatal/postnatal care) and community pharmacy counseling while developing concise, evidence-based Arabic-language messaging tailored to local misconceptions.

From a public health perspective, these results indicate a need for targeted, context-specific thyroid health literacy interventions that prioritize groups with comparatively lower knowledge, particularly men and individuals with lower educational attainment, and address domains with the greatest uncertainty, including medication-related risk factors and reproductive and postpartum thyroid issues. Feasible delivery modalities include brief counseling integrated into primary care and maternal health services, community pharmacy based education, and concise evidence based social media messaging. Intervention effectiveness should be evaluated using pre specified endpoints such as improvements in item level knowledge, symptom related care seeking intentions, and, where feasible, uptake of thyroid function testing among high risk groups.

Future studies should test interventions and explore awareness more deeply. For instance, educational interventions (brochures, media, workshops) could be evaluated in the Palestinian context. International evidence suggests these can work: in Lebanon, placing posters and pamphlets about thyroid eye disease in clinics boosted patient awareness from 16.5 to 63.5% (21). Similar trials could be run in primary care or community centers in Palestine to measure changes in knowledge and screening uptake. Additionally, longitudinal studies tracking awareness over time would be valuable, especially to see if younger generations (with more internet access) show improvements. Qualitative research is also needed to understand why knowledge is low, for example, focus groups might reveal specific misconceptions or fears (such as stigma about hormone therapy) that surveys miss. Finally, research should examine the impact of awareness on actual health outcomes: do better-informed patients present earlier or adhere better to treatment. Addressing these questions will help tailor public health strategies.

Several limitations warrant consideration. Recruitment relied on an online, non-probability sampling strategy that may introduce selection bias and limit generalizability. Our sample overrepresented women and university-educated individuals (71.3% women, 75.0% university education), whereas West Bank population statistics indicate a near-balanced sex distribution and a substantially lower proportion of adults with a Bachelor’s degree or higher (22, 23). This skew may bias overall knowledge estimates upward (or alter associations) relative to the broader population. The cross-sectional design precludes causal inference, and self-administered responses may be subject to misclassification due to guessing or variable interpretation of items. Future studies using probability-based or mixed-mode recruitment are needed to confirm population-representative estimates and to assess knowledge sources and information pathways.

### 4.1 Implications for practice and policy in Palestine

In the West Bank, access barriers and fragmented care pathways increase the value of early recognition and appropriate referral from primary care and community settings. The present item-level findings identify high-impact, feasible targets for low-cost interventions, particularly medication-related thyroid risk (such as amiodarone and lithium) and pregnancy/postpartum contexts. These gaps can be addressed pragmatically by embedding brief counseling prompts into primary care visits, antenatal/postnatal services, and community pharmacy practice, complemented by concise Arabic-language educational materials and evidence-based social media messaging.

## 5 Conclusion

Public knowledge of thyroid disorders in the West Bank was generally low, with the greatest gaps in high impact areas related to pregnancy and the postpartum period and to medication associated thyroid dysfunction. Knowledge differed across sociodemographic groups, supporting targeted approaches that prioritize men and individuals with lower educational attainment. These findings underscore the value of pragmatic, low cost interventions embedded within routine primary care, maternal and reproductive health services, and community pharmacy counseling. Messaging should emphasize symptom based indications for assessment, pregnancy and postpartum risk contexts, and medication safety with the need for clinical supervision and follow up testing. Future studies should test whether targeted education improves timely care seeking and safer medication practices using more representative sampling and implementation focused outcomes.

## Data Availability

The original contributions presented in the study are included in the article/supplementary material, further inquiries can be directed to the corresponding author.
